# 6,6′-Diethoxy-2,2′-[2,2-dimethylpropane-1,3-diylbis(nitrilomethylidyne)]diphenol

**DOI:** 10.1107/S1600536809007557

**Published:** 2009-03-06

**Authors:** Hoong-Kun Fun, Hadi Kargar, Reza Kia, Arezoo Jamshidvand

**Affiliations:** a X-ray Crystallography Unit, School of Physics, Universiti Sains Malaysia, 11800 USM, Penang, Malaysia; b Department of Chemistry, School of Science, Payame Noor University (PNU), Ardakan, Yazd, Iran

## Abstract

In the crystal structure, the title Schiff base compound, C_23_H_30_N_2_O_4_, exhibits crystallographic twofold rotation symmetry. The imino group is coplanar with the aromatic ring with an N—C—C—C torsion angle of -179.72 (9)°. An intra­molecular O—H⋯N hydrogen bond forms a six-membered ring, producing an *S*(6) ring motif. The dihedral angle between symmetry related benzene rings is 28.05 (5)°. The eth­oxy group makes a C—O—C—C torsion angle of −7.20 (16)° with the benzene ring. The crystal structure is stabilized by inter­molecular C—H⋯π inter­actions.

## Related literature

For bond-length data, see: Allen *et al.* (1987 ▶). For hydrogen-bond motifs, see: Bernstein *et al.* (1995 ▶). For information on Schiff base ligands, complexes and their applications, see, for example: Calligaris & Randaccio, (1987 ▶); Casellato & Vigato, (1977 ▶); Pal *et al.* (2005 ▶); Reglinski *et al.* 2004 ▶; Hou *et al.* (2001 ▶); Ren *et al.* (2002 ▶). For the stability of the temperature controller used for the data collection, see: Cosier & Glazer (1986 ▶).
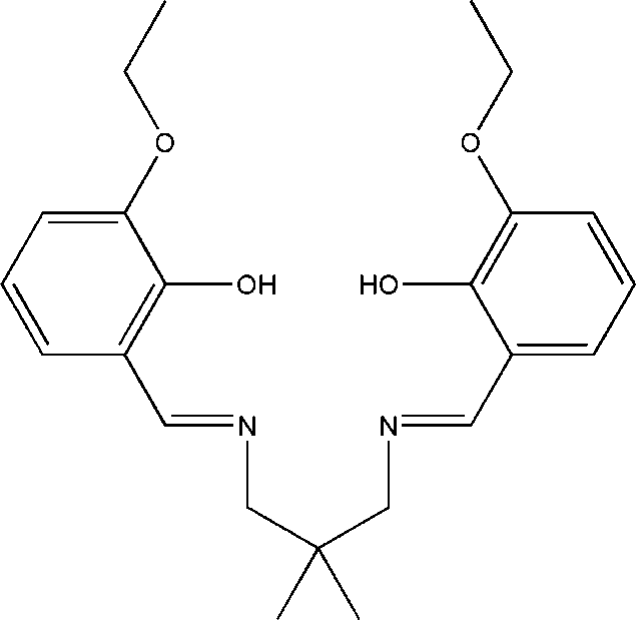


## Experimental

### 

#### Crystal data

C_23_H_30_N_2_O_4_
*M* *_r_* = 398.49
Monoclinic, 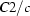

*a* = 5.6523 (1) Å
*b* = 12.9591 (2) Å
*c* = 28.3771 (3) Å
β = 91.282 (1)°
*V* = 2078.07 (5) Å^3^
*Z* = 4
Mo *K*α radiation
μ = 0.09 mm^−1^
*T* = 100 K
0.43 × 0.22 × 0.04 mm

#### Data collection

Bruker SMART APEXII CCD area-detector diffractometer
Absorption correction: multi-scan (**SADABS**; Bruker, 2005 ▶) *T*_min_ = 0.880, *T*_max_ = 0.997
21349 measured reflections
3560 independent reflections
2743 reflections with *I* > 2σ(*I*)
*R*_int_ = 0.042

#### Refinement

*R*[*F* ^2^ > 2σ(*F* ^2^)] = 0.045
*wR*(*F* ^2^) = 0.128
*S* = 1.07
3560 reflections
134 parameters
H-atom parameters constrained
Δρ_max_ = 0.42 e Å^−3^
Δρ_min_ = −0.26 e Å^−3^

### 

Data collection: *APEX2* (Bruker, 2005 ▶); cell refinement: *SAINT* (Bruker, 2005 ▶); data reduction: *SAINT*; program(s) used to solve structure: *SHELXTL* (Sheldrick, 2008 ▶); program(s) used to refine structure: *SHELXTL*; molecular graphics: *SHELXTL*; software used to prepare material for publication: *SHELXTL* and *PLATON* (Spek, 2009 ▶).

## Supplementary Material

Crystal structure: contains datablocks global, I. DOI: 10.1107/S1600536809007557/at2735sup1.cif

Structure factors: contains datablocks I. DOI: 10.1107/S1600536809007557/at2735Isup2.hkl

Additional supplementary materials:  crystallographic information; 3D view; checkCIF report

## Figures and Tables

**Table 1:** Hydrogen-bond geometry (Å, °)

*D*—H⋯*A*	*D*—H	H⋯*A*	*D*⋯*A*	*D*—H⋯*A*
O1—H1⋯N1	0.82	1.85	2.5772 (12)	147
C11—H11*A*⋯*Cg*1^i^	0.96	2.87	3.6007 (12)	133

Symmetry code: (i) 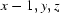. *Cg1* is the centroid of the C1–C6 benzene ring.

**Fig. 1.**
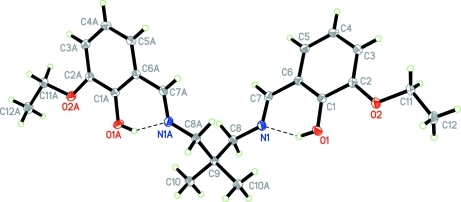
The molecular structure of the title compound with atom labels and 50% probability ellipsoids for non-H atoms. The suffix A corresponds to symmetry code (-x + 1, y, -z + 1/2).

## supplementary crystallographic information

### Comment

The condensation of primary amines with carbonyl compounds yields Schiff base (Casellato & Vigato, 1977) that are still now regarded as one of the most potential group of chelators for facile preparations of metallo-organic hybrid materials. In the past two decades, the synthesis, structure and properties of Schiff base complexes have stimulated much interest for their noteworthy contributions in single molecule-based magnetism, materials science, catalysis of many reactions like carbonylation, hydroformylation, reduction, oxidation, epoxidation and hydrolysis, *etc* (Pal *et al.*, 2005; Reglinski *et al.*, 2004; Hou *et al.*, 2001; Ren *et al.*, 2002). This is due to the fact that Schiff bases offer opportunities for inducing substrate chirality, tuning the metal-centered electronic factor and enhancing the solubility and stability of either homogeneous or heterogeneous catalysts. Only a relatively small number of free Schiff base ligands have been characterized (Calligaris & Randaccio, 1987). As an extension of our work on the structural characterization of Schiff base compounds, the title compound, is reported here.

The molecule of the title compound, (Fig. 1), has a crystallographic twofold rotation symmetry. The atom C9 lies across a crystallographic twofold rotation symmetry. An intramolecular O—H···N hydrogen bond forms a six-membered ring, producing a *S*(6) ring motif (Bernstein *et al.* 1995). The dihedral angle between the symmetry related benzene rings is 28.05 (5)°. The ethoxy group makes a torsion angle (C11—O2—C2—C3) of -7.20 (16)° with the benzene ring. The N atom is in close proximity to the H atom of the methylene group of the diamine segment, with H8B—N1 distance of 2.70 Å. The crystal structure is stabilized by intermolecular C—H···π interactions [*Cg*1 is the centroid of the C1–C6 benzene ring] (Table 1).

### Experimental

The synthetic method has been described earlier (Reglinski *et al.*, 2004), except that 3-ethoxysalicylaldehyde was used. Single crystals suitable for *X*-ray diffraction were obtained by evaporation of an methanol solution at room temperature.

### Refinement

H atom of the hydroxy group was positioned by a freely rotating O—H bond and constrained with a fixed distance of 0.82 Å. The rest of the hydrogen atoms were positioned geometrically with a riding model approximation with C—H = 0.93-0.97 Å and U_iso_(H) = 1.2 or 1.5 (C & O). A rotating group model was used for the methyl group of the ethoxy segment.

### Crystal data

**Table:**

C_23_H_30_N_2_O_4_	*F*(000) = 856
*M**_r_* = 398.49	*D*_x_ = 1.274 Mg m^−^^3^
Monoclinic, *C*2/*c*	Mo *K*α radiation, λ = 0.71073 Å
Hall symbol: -C 2yc	Cell parameters from 5303 reflections
*a* = 5.6523 (1) Å	θ = 2.9–31.8°
*b* = 12.9591 (2) Å	µ = 0.09 mm^−^^1^
*c* = 28.3771 (3) Å	*T* = 100 K
β = 91.282 (1)°	Plate, yellow
*V* = 2078.07 (5) Å^3^	0.43 × 0.22 × 0.04 mm
*Z* = 4	

### Data collection

**Table:**

Bruker SMART APEXII CCD area-detector diffractometer	3560 independent reflections
Radiation source: fine-focus sealed tube	2743 reflections with *I* > 2σ(*I*)
graphite	*R*_int_ = 0.042
φ and ω scans	θ_max_ = 31.8°, θ_min_ = 1.4°
Absorption correction: multi-scan (*SADABS*; Bruker, 2005)	*h* = −8→8
*T*_min_ = 0.880, *T*_max_ = 0.997	*k* = −18→19
21349 measured reflections	*l* = −42→41

### Refinement

**Table:**

Refinement on *F*^2^	Primary atom site location: structure-invariant direct methods
Least-squares matrix: full	Secondary atom site location: difference Fourier map
*R*[*F*^2^ > 2σ(*F*^2^)] = 0.045	Hydrogen site location: inferred from neighbouring sites
*wR*(*F*^2^) = 0.128	H-atom parameters constrained
*S* = 1.07	*w* = 1/[σ^2^(*F*_o_^2^) + (0.0621*P*)^2^ + 0.9557*P*] where *P* = (*F*_o_^2^ + 2*F*_c_^2^)/3
3560 reflections	(Δ/σ)_max_ = 0.001
134 parameters	Δρ_max_ = 0.42 e Å^−^^3^
0 restraints	Δρ_min_ = −0.26 e Å^−^^3^

### Special details

**Table:**

Experimental. The crystal was placed in the cold stream of an Oxford Cyrosystems Cobra open-flow nitrogen cryostat (Cosier & Glazer, 1986) operating at 100.0 (1)K.
Geometry. All esds (except the esd in the dihedral angle between two l.s. planes) are estimated using the full covariance matrix. The cell esds are taken into account individually in the estimation of esds in distances, angles and torsion angles; correlations between esds in cell parameters are only used when they are defined by crystal symmetry. An approximate (isotropic) treatment of cell esds is used for estimating esds involving l.s. planes.
Refinement. Refinement of F^2^ against ALL reflections. The weighted R-factor wR and goodness of fit S are based on F^2^, conventional R-factors R are based on F, with F set to zero for negative F^2^. The threshold expression of F^2^ > 2sigma(F^2^) is used only for calculating R-factors(gt) etc. and is not relevant to the choice of reflections for refinement. R-factors based on F^2^ are statistically about twice as large as those based on F, and R- factors based on ALL data will be even larger.

### Fractional atomic coordinates and isotropic or equivalent isotropic displacement parameters (Å^2^)

**Table:**

	*x*	*y*	*z*	*U*_iso_*/*U*_eq_	
O2	−0.21577 (13)	0.86647 (6)	0.42709 (3)	0.01900 (18)	
O1	0.07879 (14)	0.77817 (6)	0.36945 (3)	0.01951 (18)	
H1	0.1800	0.7580	0.3514	0.029*	
N1	0.44608 (15)	0.78978 (7)	0.31751 (3)	0.01556 (18)	
C1	0.11143 (18)	0.87962 (8)	0.37834 (4)	0.0150 (2)	
C2	−0.04483 (18)	0.92916 (8)	0.40916 (4)	0.0157 (2)	
C3	−0.01574 (19)	1.03346 (8)	0.41899 (4)	0.0180 (2)	
H3A	−0.1215	1.0665	0.4386	0.022*	
C4	0.1713 (2)	1.08960 (8)	0.39974 (4)	0.0189 (2)	
H4A	0.1897	1.1593	0.4066	0.023*	
C5	0.32737 (19)	1.04064 (8)	0.37052 (4)	0.0168 (2)	
H5A	0.4531	1.0774	0.3582	0.020*	
C6	0.29882 (18)	0.93596 (8)	0.35912 (3)	0.01445 (19)	
C7	0.46777 (18)	0.88493 (8)	0.32869 (3)	0.0151 (2)	
H7A	0.5941	0.9225	0.3172	0.018*	
C8	0.62015 (17)	0.74010 (8)	0.28797 (4)	0.0149 (2)	
H8A	0.7249	0.6980	0.3075	0.018*	
H8B	0.7150	0.7925	0.2729	0.018*	
C9	0.5000	0.67209 (11)	0.2500	0.0132 (3)	
C10	0.68697 (18)	0.60294 (9)	0.22779 (4)	0.0175 (2)	
H10A	0.7602	0.5606	0.2518	0.026*	
H10B	0.8049	0.6451	0.2134	0.026*	
H10C	0.6130	0.5597	0.2043	0.026*	
C11	−0.36178 (19)	0.90890 (9)	0.46303 (4)	0.0181 (2)	
H11A	−0.4504	0.9675	0.4508	0.022*	
H11B	−0.2647	0.9318	0.4896	0.022*	
C12	−0.5284 (2)	0.82495 (9)	0.47814 (4)	0.0215 (2)	
H12A	−0.6358	0.8522	0.5007	0.032*	
H12B	−0.4392	0.7694	0.4921	0.032*	
H12C	−0.6163	0.7998	0.4512	0.032*	

### Atomic displacement parameters (Å^2^)

**Table:**

	*U*^11^	*U*^22^	*U*^33^	*U*^12^	*U*^13^	*U*^23^
O2	0.0195 (4)	0.0172 (4)	0.0207 (4)	−0.0019 (3)	0.0086 (3)	−0.0030 (3)
O1	0.0212 (4)	0.0142 (4)	0.0235 (4)	−0.0046 (3)	0.0087 (3)	−0.0061 (3)
N1	0.0162 (4)	0.0165 (4)	0.0141 (4)	−0.0009 (3)	0.0029 (3)	−0.0018 (3)
C1	0.0177 (5)	0.0132 (5)	0.0141 (4)	−0.0009 (3)	0.0004 (3)	−0.0010 (3)
C2	0.0165 (4)	0.0159 (5)	0.0147 (4)	−0.0005 (4)	0.0022 (3)	−0.0001 (4)
C3	0.0221 (5)	0.0157 (5)	0.0163 (5)	0.0017 (4)	0.0026 (4)	−0.0015 (4)
C4	0.0249 (5)	0.0128 (5)	0.0190 (5)	0.0002 (4)	0.0010 (4)	−0.0003 (4)
C5	0.0200 (5)	0.0142 (5)	0.0164 (4)	−0.0018 (4)	0.0009 (4)	0.0012 (4)
C6	0.0164 (4)	0.0144 (5)	0.0126 (4)	−0.0006 (3)	0.0008 (3)	0.0004 (3)
C7	0.0155 (4)	0.0166 (5)	0.0134 (4)	−0.0022 (3)	0.0014 (3)	0.0017 (3)
C8	0.0132 (4)	0.0160 (5)	0.0154 (4)	−0.0011 (3)	0.0029 (3)	−0.0002 (3)
C9	0.0132 (6)	0.0131 (6)	0.0134 (6)	0.000	0.0034 (5)	0.000
C10	0.0174 (5)	0.0168 (5)	0.0186 (5)	0.0039 (4)	0.0032 (4)	−0.0005 (4)
C11	0.0194 (5)	0.0196 (5)	0.0157 (4)	0.0015 (4)	0.0051 (4)	−0.0012 (4)
C12	0.0191 (5)	0.0243 (6)	0.0213 (5)	0.0011 (4)	0.0060 (4)	0.0020 (4)

### Geometric parameters (Å, °)

**Table:**

O2—C2	1.3692 (12)	C7—H7A	0.9300
O2—C11	1.4357 (12)	C8—C9	1.5382 (13)
O1—C1	1.3506 (12)	C8—H8A	0.9700
O1—H1	0.8200	C8—H8B	0.9700
N1—C7	1.2784 (13)	C9—C10	1.5319 (13)
N1—C8	1.4568 (13)	C9—C10^i^	1.5319 (13)
C1—C6	1.4065 (14)	C9—C8^i^	1.5382 (13)
C1—C2	1.4112 (14)	C10—H10A	0.9600
C2—C3	1.3890 (15)	C10—H10B	0.9600
C3—C4	1.4039 (15)	C10—H10C	0.9600
C3—H3A	0.9300	C11—C12	1.5075 (16)
C4—C5	1.3789 (15)	C11—H11A	0.9700
C4—H4A	0.9300	C11—H11B	0.9700
C5—C6	1.4031 (14)	C12—H12A	0.9600
C5—H5A	0.9300	C12—H12B	0.9600
C6—C7	1.4601 (14)	C12—H12C	0.9600
			
C2—O2—C11	117.29 (8)	N1—C8—H8B	109.4
C1—O1—H1	109.5	C9—C8—H8B	109.4
C7—N1—C8	120.45 (9)	H8A—C8—H8B	108.0
O1—C1—C6	122.26 (9)	C10—C9—C10^i^	108.40 (12)
O1—C1—C2	118.29 (9)	C10—C9—C8^i^	110.18 (6)
C6—C1—C2	119.43 (9)	C10^i^—C9—C8^i^	108.99 (6)
O2—C2—C3	125.73 (9)	C10—C9—C8	108.99 (6)
O2—C2—C1	114.65 (9)	C10^i^—C9—C8	110.18 (6)
C3—C2—C1	119.62 (9)	C8^i^—C9—C8	110.08 (12)
C2—C3—C4	120.85 (10)	C9—C10—H10A	109.5
C2—C3—H3A	119.6	C9—C10—H10B	109.5
C4—C3—H3A	119.6	H10A—C10—H10B	109.5
C5—C4—C3	119.54 (10)	C9—C10—H10C	109.5
C5—C4—H4A	120.2	H10A—C10—H10C	109.5
C3—C4—H4A	120.2	H10B—C10—H10C	109.5
C4—C5—C6	120.77 (10)	O2—C11—C12	107.36 (9)
C4—C5—H5A	119.6	O2—C11—H11A	110.2
C6—C5—H5A	119.6	C12—C11—H11A	110.2
C5—C6—C1	119.75 (9)	O2—C11—H11B	110.2
C5—C6—C7	120.06 (9)	C12—C11—H11B	110.2
C1—C6—C7	120.16 (9)	H11A—C11—H11B	108.5
N1—C7—C6	121.55 (9)	C11—C12—H12A	109.5
N1—C7—H7A	119.2	C11—C12—H12B	109.5
C6—C7—H7A	119.2	H12A—C12—H12B	109.5
N1—C8—C9	111.29 (7)	C11—C12—H12C	109.5
N1—C8—H8A	109.4	H12A—C12—H12C	109.5
C9—C8—H8A	109.4	H12B—C12—H12C	109.5
			
C11—O2—C2—C3	−7.20 (15)	O1—C1—C6—C5	−178.44 (10)
C11—O2—C2—C1	172.54 (8)	C2—C1—C6—C5	−0.41 (15)
O1—C1—C2—O2	0.25 (14)	O1—C1—C6—C7	−0.50 (15)
C6—C1—C2—O2	−177.86 (9)	C2—C1—C6—C7	177.53 (9)
O1—C1—C2—C3	180.00 (9)	C8—N1—C7—C6	−178.59 (9)
C6—C1—C2—C3	1.89 (15)	C5—C6—C7—N1	−179.73 (10)
O2—C2—C3—C4	177.87 (10)	C1—C6—C7—N1	2.34 (15)
C1—C2—C3—C4	−1.86 (15)	C7—N1—C8—C9	−136.31 (10)
C2—C3—C4—C5	0.31 (16)	N1—C8—C9—C10	−166.89 (8)
C3—C4—C5—C6	1.21 (16)	N1—C8—C9—C10^i^	−48.07 (11)
C4—C5—C6—C1	−1.15 (15)	N1—C8—C9—C8^i^	72.16 (7)
C4—C5—C6—C7	−179.09 (9)	C2—O2—C11—C12	−178.04 (9)

Symmetry codes: (i) −*x*+1, *y*, −*z*+1/2.

### Hydrogen-bond geometry (Å, °)

**Table:**

*D*—H···*A*	*D*—H	H···*A*	*D*···*A*	*D*—H···*A*
O1—H1···N1	0.82	1.85	2.5772 (12)	147
C11—H11A···Cg1^ii^	0.96	2.87	3.6007 (12)	133

Symmetry codes: (ii) *x*−1, *y*, *z*.
